# Therapeutic Delivery Specifications Identified Through Compartmental Analysis of a Mesenchymal Stromal Cell-Immune Reaction

**DOI:** 10.1038/s41598-018-24971-2

**Published:** 2018-05-01

**Authors:** Matthew Li, Danika Khong, Ling-Yee Chin, Amy Singleton, Biju Parekkadan

**Affiliations:** 1 Center for Surgery, Innovation, and Bioengineering, Department of Surgery, Massachusetts General Hospital, Harvard Medical School and the Shriners Hospitals for Children, Boston, Massachusetts 02114 USA; 2 Harvard Stem Cell Institute, Cambridge, Massachusetts 02138 USA; 3 Department of Biomedical Engineering, Rutgers University, Piscataway, New Jersey 08854 USA

## Abstract

Despite widespread preclinical success, mesenchymal stromal cell (MSC) therapy has not reached consistent pivotal clinical endpoints in primary indications of autoinflammatory diseases. Numerous studies aim to uncover specific mechanisms of action towards better control of therapy using *in vitro* immunomodulation assays. However, many of these immunomodulation assays are imperfectly designed to accurately recapitulate microenvironment conditions where MSCs act. To increase our understanding of MSC efficacy, we herein conduct a systems level microenvironment approach to define compartmental features that can influence the delivery of MSCs’ immunomodulatory effect *in vitro* in a more quantitative manner than ever before. Using this approach, we notably uncover an improved MSC quantification method with predictive cross-study applicability and unveil the key importance of system volume, time exposure to MSCs, and cross-communication between MSC and T cell populations to realize full therapeutic effect. The application of these compartmental analysis can improve our understanding of MSC mechanism(s) of action and further lead to administration methods that deliver MSCs within a compartment for predictable potency.

## Introduction

Mesenchymal stromal cells (MSCs) are known to suppress pathologic immune responses *in vitro* and *in vivo*^1–5^. Despite widespread pre-clinical success, MSC therapy has not reached consistent pivotal clinical endpoints in primary indications of autoimmune or autoinflammatory conditions, such as graft versus host disease (GvHD)^6–11^. These inconclusive outcomes have prevented widespread adoption of human MSC therapy with outstanding issues ranging from cell lot variability, to poor *in vivo* cell persistence, to insufficient relevant cell biomarkers which are still under investigation to identify root causes of therapeutic failure^12–18^. Ongoing efforts aim to identify robust *in vitro* potency assays with high correlation to *in vivo* therapeutic effects. These potency assays, however, have not studied the sensitivity of such potency assays to *in vitro* systems parameters and what that may signify in terms of *in vivo* microenvironment delivery of MSC therapy. Beginning with a compartmental view of delivering an MSC immunomodulatory mechanism of action, we can build towards overall improvements in identifying new strategies to refine and revisit MSC therapy.

MSCs exert a large part of their immunomodulatory function in the absence of cell-cell contact through soluble factors. This indirect immunomodulation has been well studied with respect to T cell inactivation^1,19–29^. Focused studies have been critical in establishing MSC mechanism(s) of action through the identification of specific therapeutic factors. Intrinsic to the bioavailability of the MSC secretome are requirements that these factors must diffuse over a distance at a relevant concentration and persist over some specified time for therapeutic action. These time scales are critical because effector molecules are known to have relatively short half-lives on the order of minutes to an hour^30–36^. MSCs can also sense inflammatory cues which influence their secretome in an activated state^37^. Ineffective therapy has been observed *in vivo* when administered during periods of disease remission^38,39^. It is thus becoming increasingly important to evaluate how MSCs are administered, where they localize, what tissue signaling is present to activate MSCs, and what cell numbers and persistence are expected in a local compartment. A compartmental framework that accounts for the composite effects of MSCs within a defined microenvironment will increase our overall understanding on the modes of MSC success and failure.

Herein, we apply a systems level approach to specify critical attributes of MSC therapy. Studies of concentration, reaction time, reaction volume, and cellular factors were rigorously evaluated to define important specifications for an effective T cell suppressive effect by MSCs. Implications of these important reaction parameters, once presented, are discussed in greater context for the field of MSC therapy.

## Results

### Quantitative Profiling of MSC Immunosuppression

**Figure 1:**
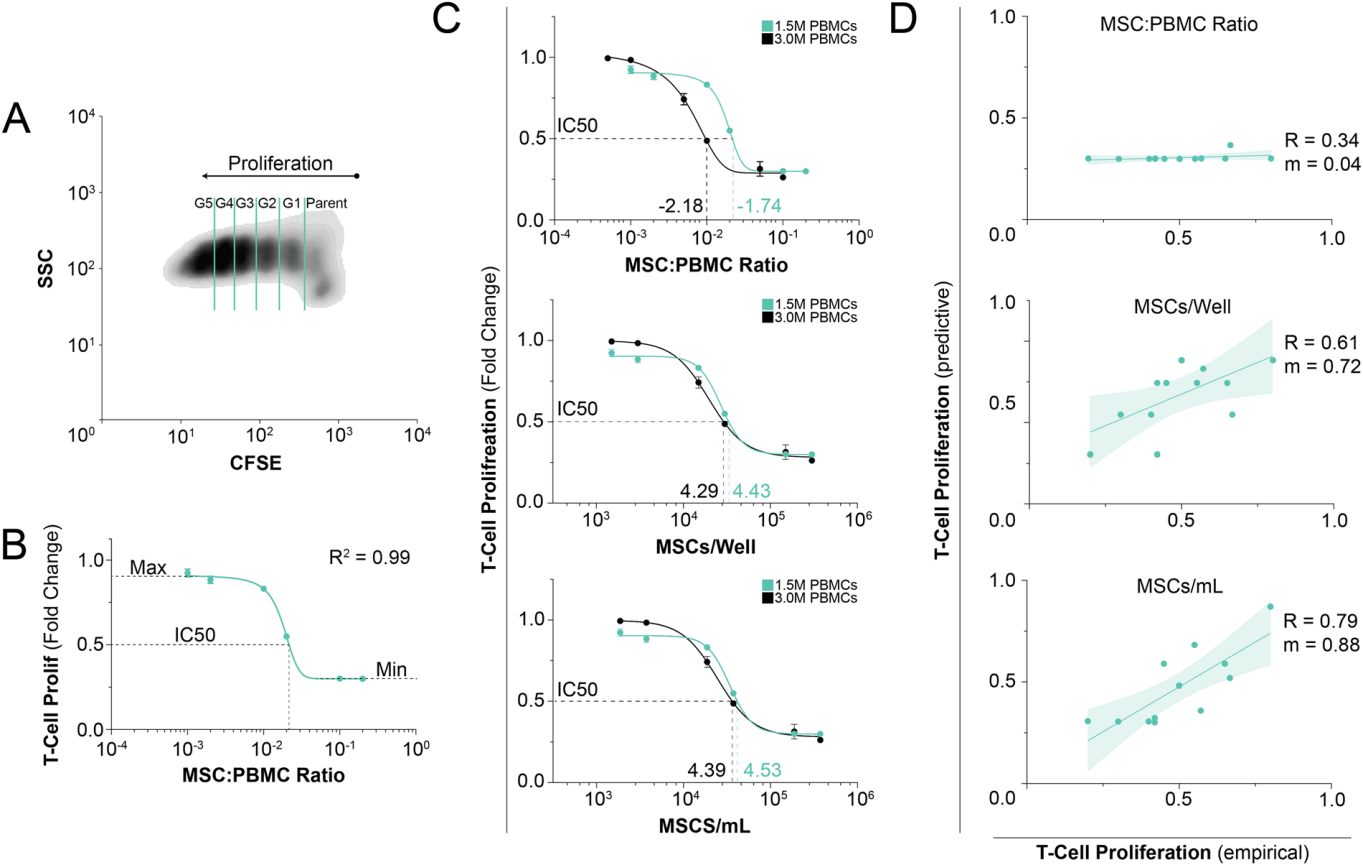
Pharmacological assessment of MSC immunosuppression with perturbation and regression analysis. PBMC proliferation was measured using flow cytometry and CFSE staining after stimulation with ConA and IL-2 for a period of 4 days. (**A**) Density plot of CFSE dilution; clear definition between proliferative generations (up to 5) is apparent. (**B**) Dose response curve of MSC suppression of T cell activation; data points represent mean +/− SD of 3 samples. Six ratios of MSCs were co-cultured with 1.5 M PBMCs to generate a full dose response curve (1:5, 1:10, 1:50, 1:100, 1:500, and 1:1000). This curve is fit by a pharmacologic dose response regression (Equation 2) with strong fit (R^2^ = 0.99). (**C**) Independent variable assessment was performed by doubling the number of PBMCs; data points represent mean +/− SD of 3 samples. (*top*) Two distinct curves form using the metric of ratio showing poor universal applicably of this metric with a nearly 3-fold difference between IC50 values. (*middle & bottom*) Implementing a cells/well and cell/mL approach, we find greatly improved agreement between these curves with a IC50 value differences less than 1.5-fold. (**D**) Each independent variable was then assessed again several matched studies to determine broad applicability using regression derived from 1C (Equation 2; regression values found in Table S1); each data point represent a distinct value from literature. Pharmacologic curves were derived from a non-linear, 4 parameter regression; correlative lines were generated using a linear regression.

Despite numerous studies that evaluated MSC dose to suppress T cell activation, a complete dose response curve that ranges multiple log concentrations with sufficient points for curve fitting has yet to be reported. Our analysis began here to evaluate the basic limits of MSC cell number on T cell modulation. PBMCs were stimulated with ConA and IL-2 for 4 days in the presence of MSCs seeded in transwells. Proliferation was assessed by CFSE and showed clear definition between T cell clone divisions (Fig. 1A). A complete response curve was achieved over a 3 orders of magnitude cell dosing (1:1000-1:5 MSC:PBMC) showing at least two points of effectiveness and ineffectiveness (Fig. 1B**)**. We find comparability between 3 separate PBMC donors demonstrating broad applicability of these findings (Fig. S1). These data strongly fit a classic dose response regression curve (Eq. 2) leading to opportunities to extract parameters to describe a MSC-T cell interaction at a systems level. The half maximal inhibitory concentration (IC50) was also extracted (MSC:PBMC ratio of 0.018). The IC50 can be an important metric to compare potency across MSC cell lots, donors, and in specific environmental conditions. MSC immunomodulation was also found to be to cell-specific (Fig. S2). Liver (HepG2) and endothelial (EA.hy296) cells lines enhanced proliferation while dermal fibroblasts (NHDF) had an inferior suppression of T cells compared to MSCs^40^. This cell specificity supports the uniqueness of bone marrow MSC immunomodulation.

A corollary to this initial dose study was defining a standard independent variable. Several reports have evaluated *in vitro* MSC doses and report results in terms of the ratio of MSCs to PBMCs or the number of MSCs per well. Our ancillary study evaluated these forms of data reporting and the sensitivity of results using each independent variable set. An MSC:PBMC coculture was established at 2 different PBMC numbers that were input into the system. When comparing between 1x and 2x PBMCs, we posit that a robust independent variable should demonstrate roughly collinear dose response curves and be insensitive to system conditions. However, there was a large discrepancy when plotting the data using “MSC:PBMC ratio” with a 2.75-fold difference in IC50s between the two curves as a standard method to cross-compare bioassay results (Fig. 1C, top). Variables of cells/well and cells/volume both demonstrate a 1.38-fold difference in IC50s which were more durable as an independent variable than ratio (Fig. 1C, middle and bottom, respectively). These results suggest the use of cell concentrations to better report data and to improve cross-study comparisons.

By standardizing the bioassay system and reporting, there existed the potential that results could have predictive value when applied to other studies with the same variables. Eight prior studies were identified in the literature that matched the *in vitro* transwell conditions used herein (Table S1). Predicted proliferative values were calculated using regression equations that fitted our data and empirical values from literature (MSC number, PBMC number, well size/volume, Table S2). Predicted values were plotted against experimental results from published results (Fig. 1D). The use of MSC:PBMC ratio as an independent variable showed poor correlation and low sensitivity. Ratio values from the literature did not span the exponential portion of our regression curve which explained the flatline behavior. The prediction of experimental results was improved by the use of MSCs/well and even further with the use of MSCs/mL. These results demonstrate, for the first time, the ability to predict results of MSC interactions between studies and the importance of reporting experimental parameters to help standardize systems under study.

### Secretome Characterization of PBMC Response to MSC Immunosuppression

**Figure 2:**
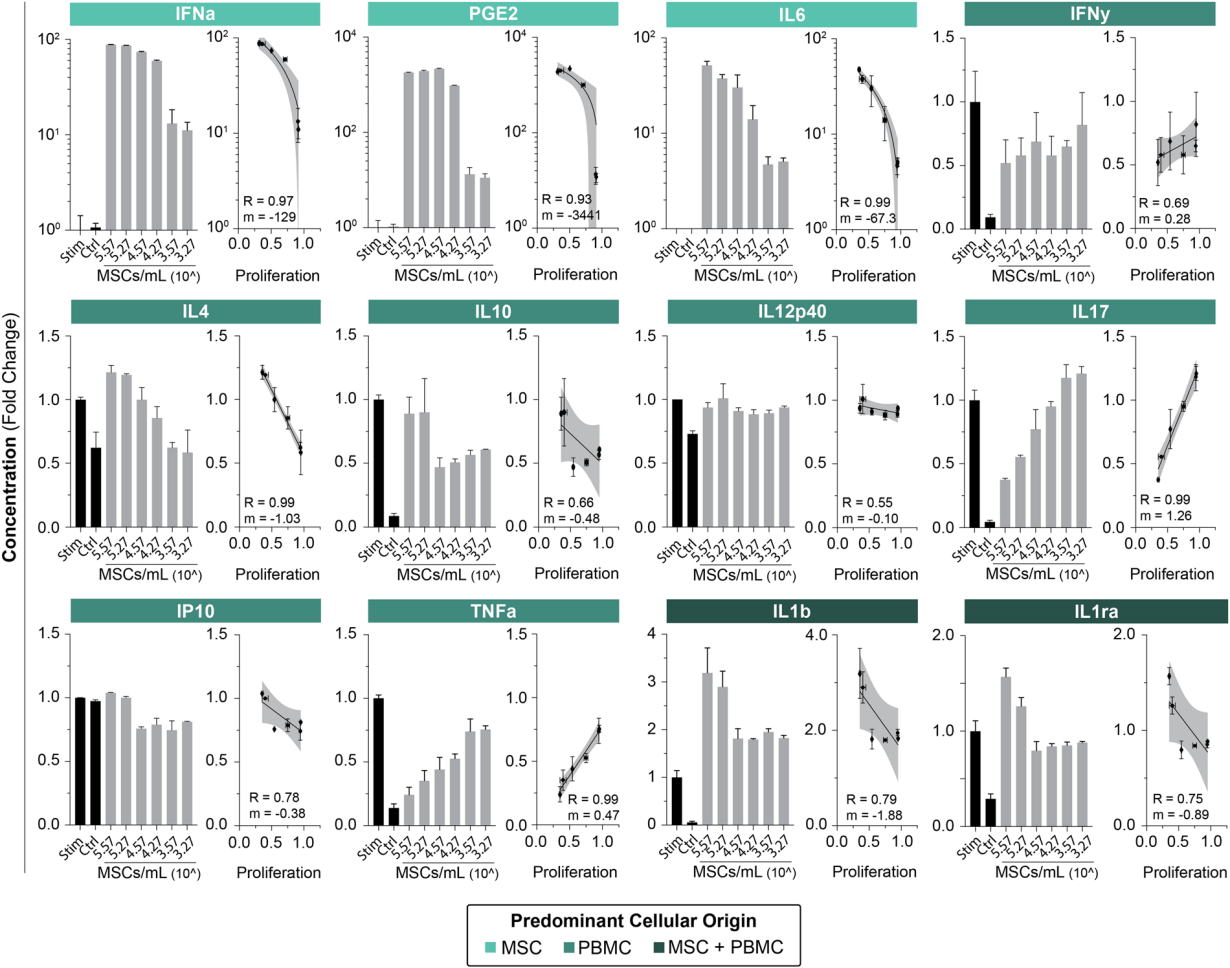
Biochemical profiling of PBMCs in response to MSC immunomodulation. PBMC proliferation was attained through stimulation with ConA and IL2 for a period of 4 days. Cytokines are binned based on their predominate origin: MSC, PBMC, or both. Bar graphs of normalized cytokine secretion versus cell concentration; bar graphs and data points represent mean +/− SD of 3 samples. MSC derived factors (IFNa, PGE2, and IL6) are highly correlated with MSC numbers. We further find that MSCs broadly downregulate secretions of pro-inflammatory cytokines (IFNy, IL17, TNFa) while promoting anti-inflammatory factors (IL4, IL10). Factors secreted by both populations (IL1b and IL1ra) demonstrate a stepwise change with moderate correlation. Correlative lines were generated using a linear regression.

The primary endpoint of the study, namely T cell suppression by MSCs, was expected to associate with changes in the biochemical composition of the compartment as secondary endpoints, Fig. 2. A corollary to this compositional change was the state of T cell activation. We unsurprisingly find that MSC immunosuppression results in reduced CD4 and CD8 proliferative generations as well as a strongly correlated downregulation of classic CD38 and CD25 activation markers (Fig. S3). MSC:PBMC assays were sampled for multiplex cytokine analysis to bin molecular biomarkers that associated with a known suppression result with their predominant origin (PBMC, MSC, or both). In the MSC group, strong correlations exist at high sensitives (IFNa, PGE2, and IL6). These results fall in line with expectations given their direct correlation with MSC numbers. High MSC concentrations results in noticeable immunosuppression and promotion of an anti-inflammatory state, which is contrary to the high expression of inflammatory factors: IFNa and IL6. These two factors are thus unlikely to be significant contributors to MSC effect or T cell proliferation *in vitro* and serve as poor analytical markers of therapeutic efficacy.

An inverse correlation with MSC numbers and PBMC pro-inflammatory cytokines was observed (IFNy, IL17, IP10, and TNFa). Anti-inflammatory cytokines were directly correlated with MSC suppression (IL4, IL10). IL12p40 remains roughly unchanged. This supports findings in literature that demonstrate MSC effect through immunologic shifts from a Th1 to a Th2 immune response profile.

We find a moderate inverse correlation in factors secreted by both cell types (IL1b, IL1ra. These factors are more complex to assess and have a noticeable step-like expression function. We believe that this step-wise transition represents a sharp drop off of MSC contribution while the plateau is a sustained activated PBMC response.

### Co-Culture Duration with MSCs is Critical to Control for Immunosuppression

**Figure 3:**
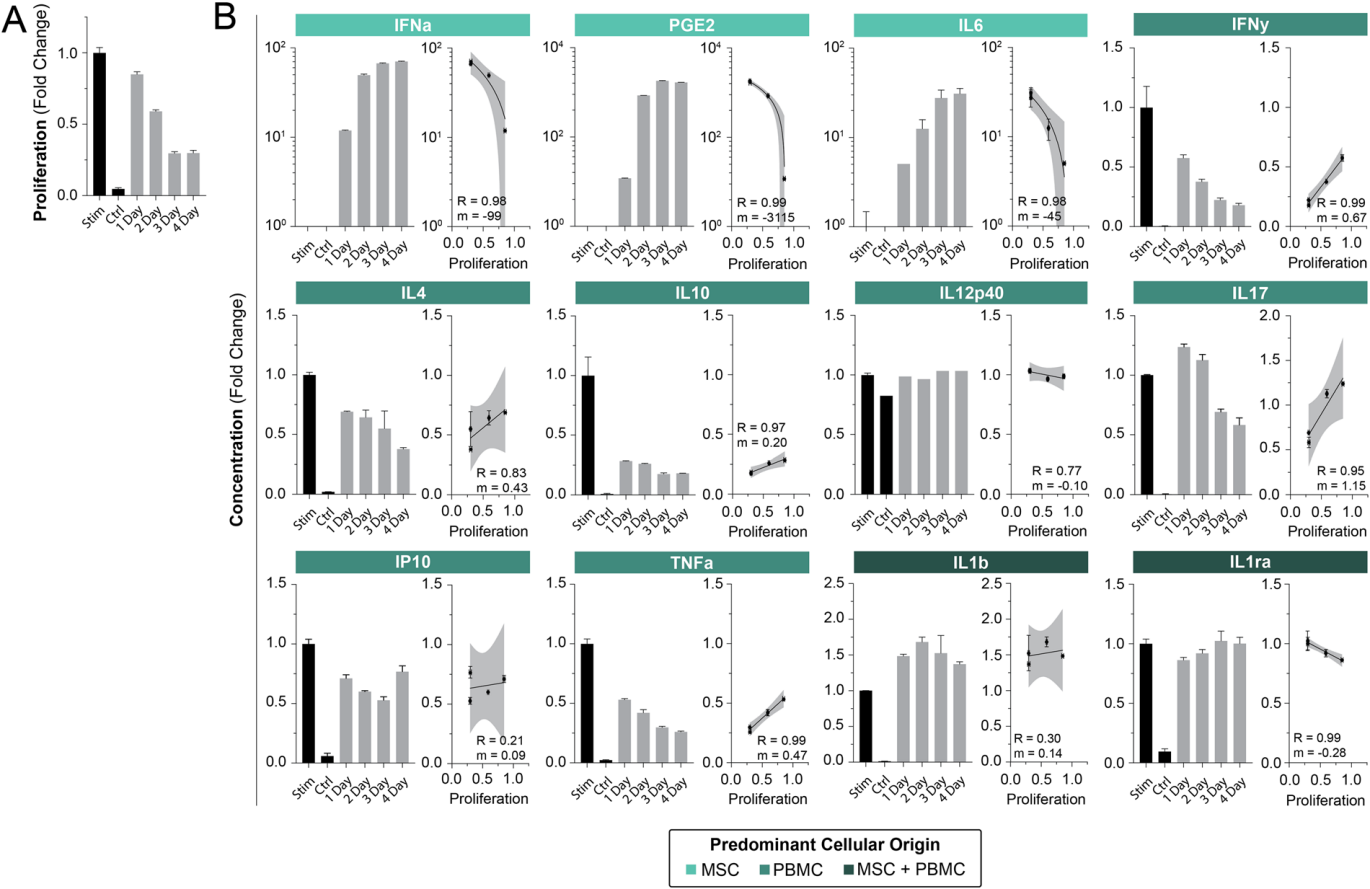
PBMC culture duration with MSCs is critical to overall immunosuppressive effect. PBMC proliferation was attained through stimulation with ConA and IL2 for a period of 4 days. MSC transwell inserts were removed after 1, 2, and 3 days co-culture initiation to time duration. Proliferation was measured through flow cytometry and CFSE staining; bar graphs and data points represent mean +/− SD of 3 samples. (**A**) Bar graphs of normalized proliferation versus time exposure to MSCs. (**B**) Bar graphs of normalized cytokine secretion versus time exposure to MSCs. Investigating cytokine profiling, we see concordant associations seen in previous sections. Longer exposure to MSCs results in greater suppression of inflammatory cytokines and vice versa for short exposures. Correlative lines were generated using a linear regression.

The interaction time, or duration, that PBMCs are exposed to MSCs is an important variable that has yet fully explored as a therapeutic specification. Prior studies have cocultured both populations for the entire assay and it is thus unknown the actual interaction time specification for immunosuppression to take effect. Duration was tested by culturing PBMCs in the presence of MSCs for 1–4 days in our proliferation assay. We observe that a full suppressive effect is realized by at minimum 3 days of co-culture, Fig. 3A. Suppression occurred at 1 and 2-day co-culture at reduced effect. This indicates that a significant time commitment is required to realize the full potency of MSCs on activated T cells.

In the previous section, we observe that MSC dose is inversely correlated with release of pro-inflammatory cytokines. We posit that, as a tangent to dose, time duration will also be inversely correlated - longer time duration will result in reduced inflammatory cytokines. By performing these parametric studies, we may now begin to assess driving factors for MSC immunomodulation of T cells *in vitro*.

MSC predominant factors rise with co-culture duration indicating an accumulation of factors (Fig. 3B). These factors also demonstrate strong inverse correlation with PBMC proliferation with high sensitivity (IFNa, PGE2, and IL6). As alluded to earlier, it is unlikely that IFNa or IL-6 are driving forces in MSC effectiveness as their pro-inflammatory nature are contradictory to the anti-inflammatory effect MSCs have on immune populations. PGE2, however, has been hypothesized as a contributing factor to MSC therapy and appears to be a potent indicator of MSC effect in our hands.

PBMC pro-inflammatory cytokines follow an anticipated trend of decreased secretion over time with strong immunoproliferation correlation (IFNy, IL17, and TNFa). We also see a similar trend with anti-inflammatory cytokines (IL4, IL10). This begins to demonstrate that these particular anti-inflammatory factors may have little effect in this model system. IL12p40 once again shows moderate correlation and poor sensitivity. IP10 shows ambiguous results with poor correlation. Factors secreted by both cells demonstrated mixed findings with weak and ambiguous results with no clear trend (IL1b) and weak trends with good correlation (IL1ra). Akin to IFNa and IL6, IL1b, a notable pro-inflammatory cytokine, demonstrates high levels at a therapeutic MSC dose which likely indicates this cytokine as a poor indicator of system effectiveness. Furthermore, IL1ra, a known anti-inflammatory factor^41^, appears to have little effect in this system.

We now begin to see a picture of candidate factors that may be implicated in the identification of systemic MSC efficacy. Based on the results thus far, PGE2, IFNy, IL17, and TNFa are of interest.

### Reaction Volume is Implicit in MSC:PBMC Communication

**Figure 4:**
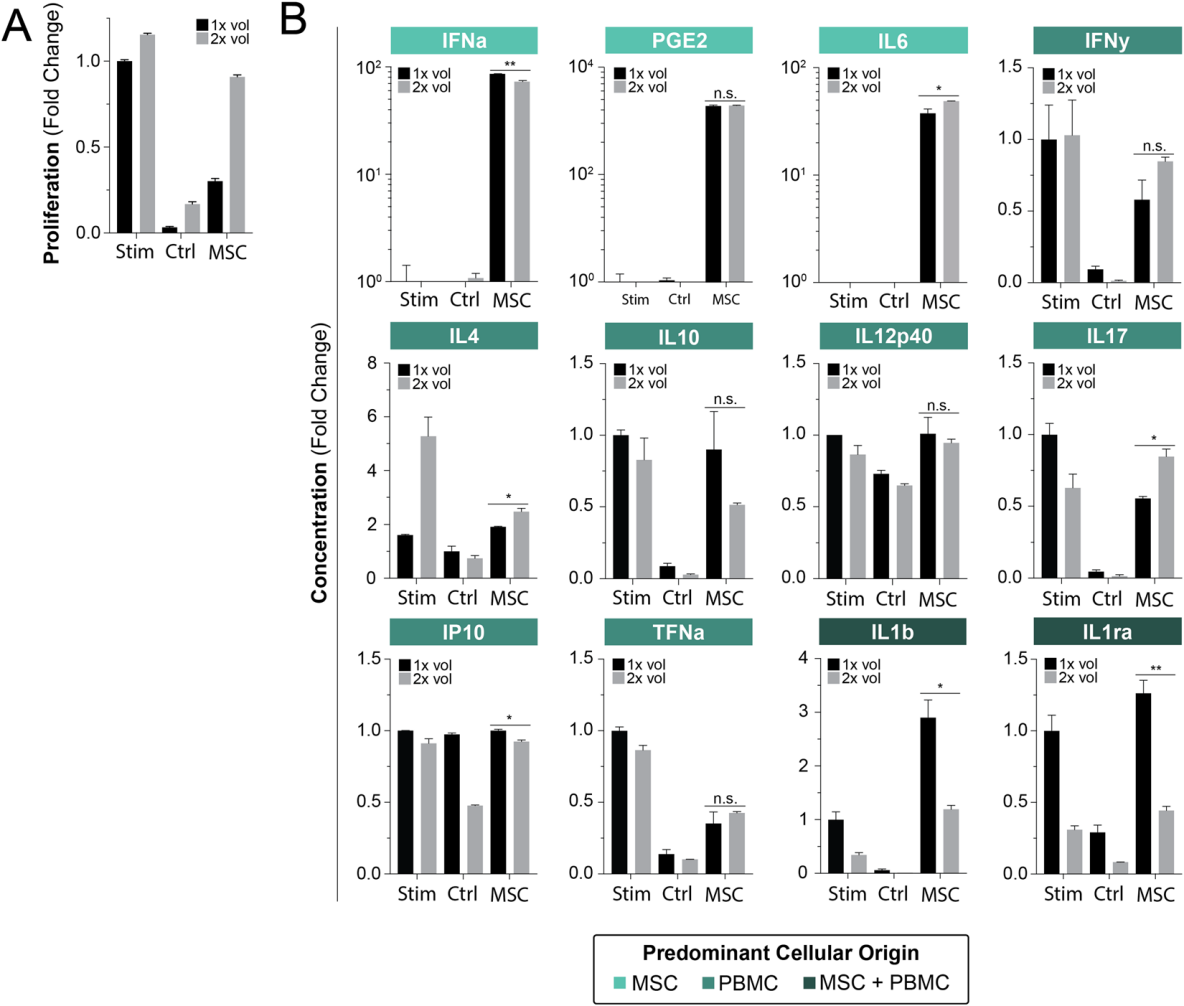
Volume is an implicit microenvironment factor driving MSC potency. PBMC proliferation was attained through stimulation with ConA and IL2 for a period of 4 days. Proliferation was measured through flow cytometry and CFSE staining; bar graphs represent mean +/− SD of 3 samples. **(A**) Bar graphs of normalized proliferation versus culture volume conditions. (**B**) Bar graphs of normalized cytokine secretion versus culture volume conditions. Two-group significance comparisons were performed with a student’s T-test; n.s., no significance; *P ≤ 0.05; **P ≤ 0.01; ***P ≤ 0.001.

Transwells inherently limit communication between co-cultured cells to secreted factors within a defined volumetric compartment. The concentration of these secreted factors as a function of compartment volume was next explored using FMEA. Doubling the volume was found to drastically reduce the immunosuppressive properties of MSCs by 3-fold (Fig. 4A). Interestingly, we also observed general rises in overall proliferation in both the stimulated control and unstimulated groups. This may speak to the dilution PBMC derived autocrine factors that innately keep growth and activation in check (i.e. IL10)^42,43^.

Cytokine results were not found as a 1:1 reflection of a dilutional volume change (Fig. 4B). MSC factors demonstrated three distinct changes: IFNa decreased, PGE2 remained unchanged, while IL6 increased. The most interesting result is the lack of PGE2 change, a factor that has been shown to comprise a functional portion of the MSC secretome. We thus observe a loss of MSC function without a decrease in PGE secretion indicating a lack of PGE2 effectiveness in our proliferation assay.

As indicated by our earlier results, we expect to find elevated inflammatory PBMC factors at higher volumes due to reduced MSC effect. PBMC factors demonstrate the following elevated factors (Fig. 4B): IFNy, IL4, IL17, and TNFa. We further find the following factors decrease: IL10, IL12p40, and IP10. IL10 is a known anti-inflammatory and thus has potential correlation with reduced concentration, however results are not significant. Further changes in IL12p40 and IP10 are low and unlikely to be of causative nature. We once again observe some the usual suspects of IFNy, IL17, and TNFa as markers of MSC effectiveness.

We then observe that some of the largest decreases occur in factors secreted by both populations (Fig. 4B): IL1b and IL1ra. We observe that these factors appear to have the most direct response to a two-fold dilution by nearly halving their respective fold change secretions.

### Cross Communication of Soluble Factors Is Required for MSC Immunosuppression

MSC licensing has been shown to enhance MSC function^37^. We herein, for the first time, demonstrate that reduced cell numbers and overall blockade of PBMC factors abrogates MSC efficacy. We first observed that a 4-fold reduction in PMBC numbers causes a 2-fold decrease in MSC efficacy, Fig. S4, likely due to insufficient PBMC factors to effectively license MSCs. In this sense, MSCs are unlike traditional pharmacologic agents and more akin to prodrugs in which some process is required to convert them into an active state. We introduce Brefeldin A (BA), a protein transport inhibitor, to block communication between MSCs and PBMCs to assess cellular communication. We first validate that BA treatment, near fully abolishes the immunosuppressive effects of MSCs, Fig. S5. While not surprising, this demonstrates that a significant portion of MSC effect can be derived from secreted factors.

**Figure 5:**
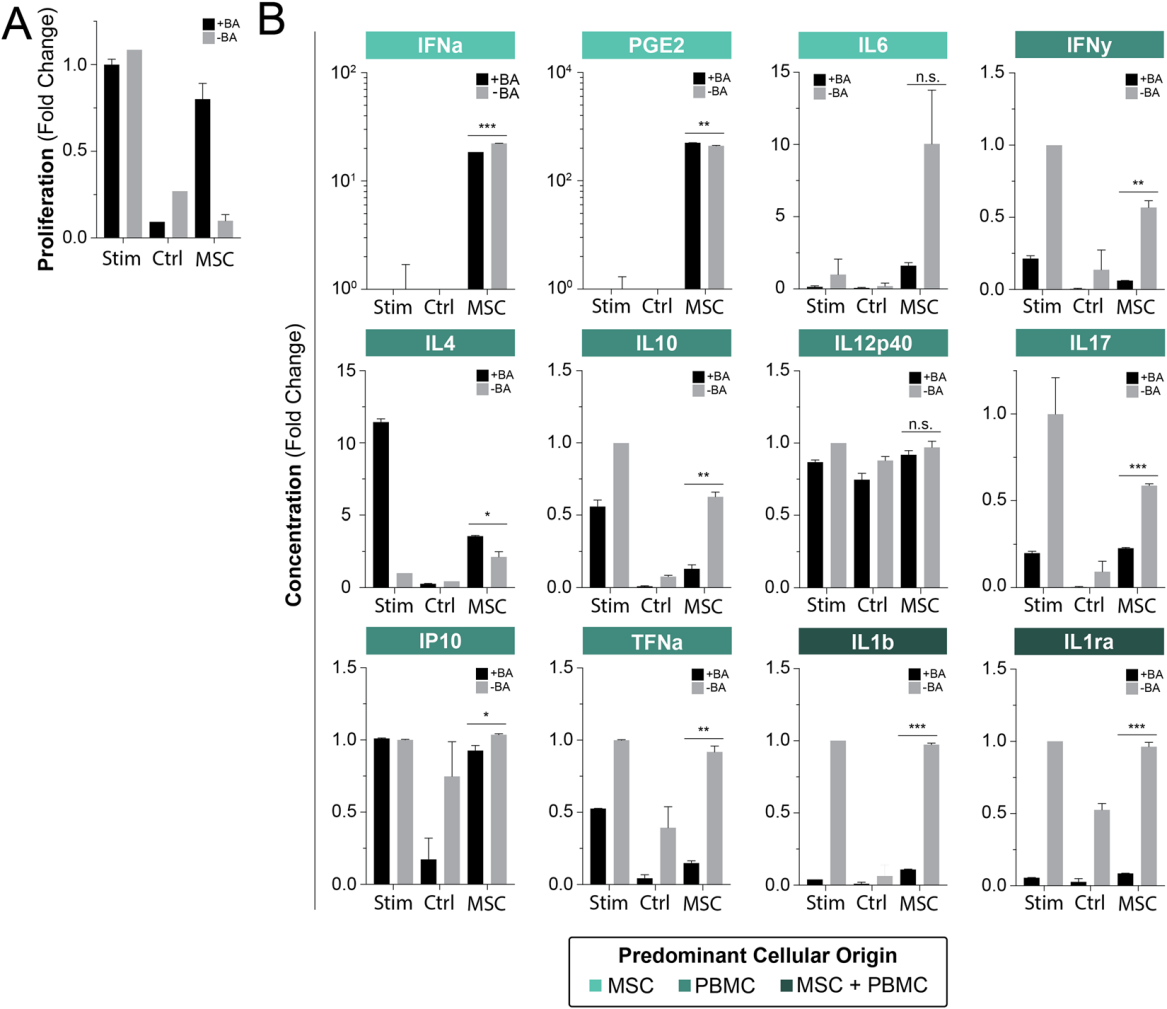
MSC:PBMC cross communication is critical as shown by the use of a protein transport inhibitor. PBMCs were treated for 24 hours with Brefeldin A prior to the start of co-culture; +BA indicates with brefeldin A pretreatment, -BA indicates without brefeldin A pretreatment. PBMC proliferation was attained through stimulation with ConA and IL2 for a period of 4 days. Proliferation was measured through flow cytometry and CFSE staining; bar graphs represent mean +/− SD of 3 samples. (**A**) Bar graphs of normalized proliferation versus Brefeldin A conditions. (**B**) Bar graphs of normalized cytokine secretion versus Brefeldin A conditions. Two-group significance comparisons were performed with a student’s T-test; n.s., no significance; *P ≤ 0.05; **P ≤ 0.01; ***P ≤ 0.001.

We then reversed the assay and treated PBMCs with BA to confirm the need for MSC licensing through PBMC secreted factors. We find that BA treated PBMCs, co-cultured with MSCs, still retain a significant ability to proliferate versus untreated PBMCS indicating a drastic reduction in MSC immunosuppresiveness, Fig. 5C. This confirms the need for communication between these cell population to license MSCs.

We once again see statistically significant changes in MSC factors (IFNa, PGE2), Fig. 5D, however with minimal absolute changes. Contrary to expectations, we see a rise in PGE2 with reduced MSC efficacy compared to untreated BA group. We observe a large change in IL6, however not significant. These results further remove weight from these factors as having therapeutic or diagnostic applicability in this system.

We unsurprisingly see significant decreases in PBMC factors: IFNy, IL17, and TNFa, Fig. 5D. This is highly concordant with increased proliferative capacity and may likely speak to reduced MSC licensing. We also observe an associated drop in anti-inflammatory IL10. We interestingly see an increase in IL4. Again, IP10 and IL12p40 demonstrates poor association and thus not likely of much intrinsic importance in our findings. We also observe significant decreases in both IL1b and IL1ra, Fig. 5D. These decreases are likely a reflection of the direct loss of PBMC secretions as well as reduced MSC production due to insufficient licensing.

## Discussion

A systematic approach helps to identify elements of design, manufacturing, or product components that may contribute to possible therapeutic failure modes. This study applied such an approach to MSC therapy with a biological focus on the compartment of MSC action with respect to immunomodulation to better define governing microenvironment interactions. These results have ramifications on the field at large that is still currently struggling to validate *in vivo* human efficacy in large trials that harmonize with potency assays and robust reference standards. The ability to create such a framework from *in vivo* efficacy to reference standards will instill greater confidence in cell therapy products and minimize variability in MSC products across all users.

The results herein are from a singular MSC donor. Variance in MSC donor responses is critical to measure for the release of a MSC therapy lot. As such the ISCT has released a perspective on immune functional aspects in the context of MSC potency release criterion^44^. In this perspective, they acknowledge the use of classic immunomodulatory assays, yet raise the valid point of potential heterogeneous outcomes from mixed PBMC populations. We tested two additional PBMC donors at 3 different MSC:PBMC ratios and found non-significant variation between their responses indicating good reproducibility in our hands.

The microenvironment is highly relevant to MSC therapy partly due to their nature of homing and engrafting to sites of inflammation and injury and thus acting upon cells and tissues locally. Circumventing this, investigators are also directly injecting MSCs into these sites to avoid complications associated with intravenous MSC delivery such as cell accumulation in regions distal to the site of injury. The *in vitro* co-culture system in our study models a local microenvironment volume of roughly 1 cm^3^; which is on the order of magnitude of local lesions in myocardium and cartilage for which MSC therapy is being assessed. A distinct challenge in a studying paracrine effects of MSC systemic infusions is to have a precise and spatial detection of MSCs in a tissue bed over time. Furthermore, additional diffusional barriers exist including: local interstitial flow rates, natural degradation and uptake of factors by other cells, binding of factors to insoluble matrix proteins. Thus, if a particular condition fails in a significantly smaller microenvironment condition, it points to the notion that it is highly unlikely to work in a more diffuse, global paracrine model. Such *in vitro* modeling efforts becomes valuable to the community as a robust, cursory screening tool towards defining biodistribution criteria for effective therapeutic MSC action.

**Figure 6:**
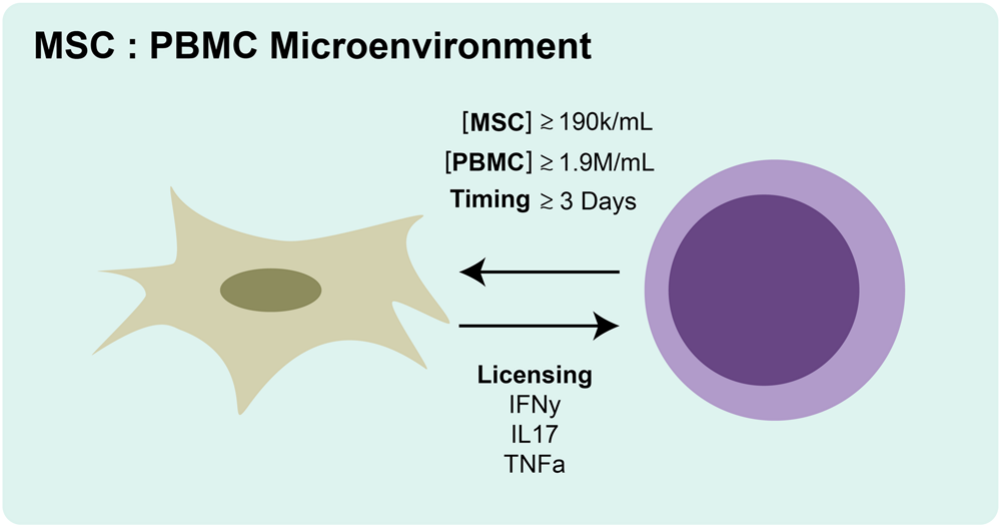
System specifications of MSC:PBMC microenvironment for an effective immunomodulatory outcome. We find that minimum MSC and PBMC are required for therapeutic function due to secreted factors and the need for MSC licensing. Furthermore, significant time exposure to MSCs is required. In our hands, we discover that the three most relevant factors for overall effect (from licensing through to overall downregulation) are IFNy, IL17, and TNFa. These findings, while performed *in vitro*, are quite informative are suggest the need for significant time exposure and an appropriately inflammatory environment in order to coax a therapeutic effect.

A pharmacologic framework of such a compartment where MSCs interact with an activated T cell population unveiled governing properties of cellular concentration, reaction volume, timing, and crosstalk that specify important system criteria to be met for effective immunomodulation (Fig. 6). The composite of two variables (dose and exposure) contributes to a parameter known as area under the curve (AUC). Akin to their use in pharmacology, optimization of these parameters will yield improved therapeutic potency.

A widely-used T cell activation assay was used as a reproducible bioassay of MSC immunomodulation. T cell suppression was observed within a therapeutic range of MSCs and found to be cell-specific. It is noteworthy to observe enhancement of T cell proliferation by other cell types, a phenomenon that could find use in the *ex vivo* growth of T cells for leukocyte therapies. *In vitro* testing also identified clear secondary endpoints of molecular biomarkers (IFNy, TNFa, and IL17) and immunophenotypic changes in T cells that can be clearly tracked as a pharmacodynamics response to MSC therapy^38,45–47^. Such pharmacodynamic responses have been observed in mouse models, though human response data is lacking and may reflect ineffective scaling of MSC dosing to larger subjects. An independent variable of MSCs per volume (cells/mL) was found to be a more robust independent variable than MSC:PBMC ratio and opened up opportunities to use regression fitting of dose response curves to predict outcomes when applied to matched studies. Standardizing the bioassay results to a scalable variable with defined units may aid in translating *in vitro* results to *in vivo* studies with logical dose justification. The T cell effects observed are based on a polyclonal stimulation method and highlighted an attenuation of pro-inflammatory populations (i.e. Th1 and Th17) by MSCs confirmed through cytokine analysis^1,2,48^. In contrast, MSCs promoted an anti-inflammatory (Th2) profile which is in line with studies focused on the generation of regulatory T cells^2,49–51^. The customization of this assay to explore specific T cell subsets, disease-specific stimulants, and/or patient cells can offer a more clinically relevant view of MSC therapy for a given application. Furthermore, tissue-based T cells are intrinsically different than peripheral blood T cells and may inform expectations about immunomodulation for approaches where MSCs are targeted to specific tissue beds.

Major system components of the MSC-T cell compartment of action were further explored. The interaction time for this cell-cell interaction to take place was next under study. A minimum of 3 days of MSC exposure was required for a maximal effect. Other groups employing MSC co-culture with macrophages also note a co-culture period of 3–4 days which falls in line with our findings and suggests other MSC-immune cell interactions may have similar time scales^52^. It is worthy of extrapolating this result to *in vivo* testing of MSCs. Intravenously administered MSCs tracked by sensitive methods do not seem to persist *in vivo* with an estimated half-life of 24 hours in mouse models^15,16^. The viability of MSC transplants can further be impacted by co-administered agents as well. In humans, an early clinical trial that tracked the blood bioavailability of MSCs reported only 14% of patients with detectable levels of circulating MSCs after only 1 hour post-infusion^53^. The time scale of a viable MSC mass remaining in a target tissue bed based on these studies and in reference to our *in vitro* analysis would suggest that an effective duration of interaction is unlikely by current intravenous administration routes. Exploring more rigorous methods to detect MSCs in human patients over time and body compartment will help put *in vitro* testing into a clinical reality.

Biodistribution volume is an intrinsic property that was explored and also revealed important insights. Simply stated, the volume can directly affect the overall concentration of delivered MSC soluble factors. We observed that MSC suppression of T cells was extremely sensitive to volume, where increasing the volume by a 2x nearly eliminated the suppression outcome. This highlights an underexplored area regarding the volume that MSCs encounter *in vivo*. Depending on biodistribution and selective tissue engraftment efficiency, different numbers of MSCs may be expected per a given tissue bed volume and interact with a certain density of resident T cells therein. Adapting multi-compartment distribution pharmacokinetic models to account for tissue selectivity, tissue volume, and resident T cell densities may aid in more precise determination of effective or ineffective local suppression outcomes. Volume can also be perceived from a spatial understanding. The physical microenvironment has finite boundaries over which soluble factors or vesicles must traverse. Therapeutic factors must thus not only reach their target in a timely manner, but do so at a relevant concentration. This becomes exponentially more challenging the farther apart effector and responder cells in three dimensional spaces. In practice, researchers and clinicians are implementing locally administered to improve the pharmacologic length scales that these cells will be required to work over compared so systemically administered MSCs.

A unique aspect of cell therapy is the ability to leverage intrinsic sensing mechanism to respond to local environments in a more physiological way. Naïve MSCs may only develop an immunosuppressive phenotype in response to inflammatory cues; a phenomena referred to as “licensing”^37,54^. The responsiveness of MSCs is directly linked to the disease state of the recipient as demonstrated by reduced efficacy when administered in states of remission^38,39^. Our study confirms a loss of MSC immunomodulation when PBMCs were pre-treated with BA, thereby disrupting PBMCs’ ability to secrete molecules to activate MSCs. This study confirms the need for dynamic crosstalk with PBMCs to induce a suppressive outcome within a local compartment. IFNy is considered an important mediator with support from inflammatory cytokines: IL-1b, IL-17, and TNFa^55–57^. This process has been employed *in vivo* wherein investigators have successfully pretreated MSCs with these inflammatory to enhance their function^55–57^. Yet, we found little to no benefit of pre-treating MSCs with those inflammatory cues at therapeutic and sub-therapeutic MSC concentrations *in vitro* (Fig. S6). These licensing cues may be more relevant for other MSC-immune interactions or a combination of stimulants may be required under a certain dynamic of cytokine signaling. Biomarker tests measuring levels of critical inflammatory mediators of MSC licensing would be of great utility as a screening tool as to which subjects might be most responsive to MSC therapy. For instance, morphological changes in IFNy stimulated MSCs has demonstrated potential as a screening mechanism for MSC potency^58^. Not only will this promote improved MSC potency, but will minimize any potential adverse reactions by administering cells to likely non-responsive patients. Retrospective analyses on any potentially saved patient samples in an attempt to correlate cytokine levels with outcomes would be of high value.

Accordingly, revisiting administration strategies in development that aim to increase MSC exposure are worth evaluation given these identified constraints. The tactic of simply delivering more cells by intravenous administration routes may not overcome these issues due to degradation and maximal dose limits. This approach is still hampered by a relevant cell concentration at a target tissue compartment as well as a duration of effective MSC-T cell interaction for modulation outcomes^59^. Overexpression of a pro-survival gene such as AKT1 to increase *in vivo* cell persistence^60,61^ can impact target tissue MSC concentration and duration with a deeper study of these parameters. Prolonged MSC survival, however, runs the risk of adverse cell differentiation and potential neoplastic support^62,63^. Numerous studies are using local delivery of cells to assure target MSC doses to impact local microenvironment interactions^64–67^, which has found success in fistulizing Crohn’s Disease. Systemic therapy may not be served by this administration method. Methods to encapsulate MSCs and minimize local immune clearance without detriment to their function^68^ offer promise, though more rigorous studies are needed to determine if MSC viability is an issue for local delivery. Encapsulated MSCs are much larger structures making intravenous infusion routes dangerous for pulmonary toxicity concerns. *Ex vivo* approaches remove the need for MSC infusions while promoting a favorable, controlled microenvironment in terms of dose and duration. Coculture of MSCs and autologous immune cells *in vitro* prior to reintroduction of conditioned immune cells is one such method, though the time frame of coculture is very transient and immunological effects stated warrant reproduction by others^52,69^. Another method is through the use of extracorporeal devices which can be directly connected to subjects for acute care and has been shown to effectively reverse hepatic failure in rats^70^. While promising, these methods add complexity and invasiveness in terms of administration as well as additional device costs that must be balanced against the benefits of MSC therapy^71,72^.

An additional variable that becomes important is the interaction of MSCs with certain cell types in relation to the primary mode of intravenous delivery; these cells are predominantly endothelial cells and platelets. Studies have demonstrated a positive effect between MSCs and ECs in supporting angiogenesis and maintaining endothelial integrity.

However, *in vitro* studies have also demonstrated enhanced osteogenic differentiation in the right conditions which will lead to reduced efficacy if occurring *in vivo*^73^. Platelets may play a more detrimental role as it has been demonstrated that activated platelets, as can be witnessed at sites of injection, can interfere with MSC homing^74^.

MSCs are a dynamic cell therapy that function in concert with their surrounding microenvironment. We herein demonstrate that, within a microenvironment reaction compartment, the interaction between MSCs and peripheral T cells can be governed by volumetric, temporal, and cell-cell communication signaling kinetics that define specifications and failure modes for effective immunomodulation. The application of these compartmental analysis can improve our understanding of MSC mechanism(s) of action and further lead to administration methods that deliver MSCs within a compartment for predictable potency.

## Methods

### Human Cell Cultures

Mesenchymal stromal cells were isolated from whole bone marrow aspirates obtained from a single healthy donor (Lonza, Basel, Switzerland). We acknowledge the existence of MSC donor variability, however, in depth study of this is outside the scope of this work. Per the Lonza technical specifications sheet, all marrow donations were performed with informed consent with their Donor Program currently approved by a commercial institutional review board. All methods involving these samples were performed in accordance with institutional and biosafety regulations. The aspirate was firstly diluted 1:10 in in ACK lysis buffer (BioLegend, Dedham, MA) to remove contaminating erythrocytes. After a 5-minute incubation, an equal volume of phosphate buffered saline (PBS, Sigma, St. Louis, MO) was added and the samples centrifuged at 400 g for 10 minutes at 2–8 °C. The waste supernatant was aspirated off and the pellet was then resuspended in complete medium and again centrifuged at 400 g for 10 minutes at 2–8 °C. Complete media was composed of: Alpha-MEM (Sigma, St. Louis, MO), 10% fetal bovine serum (Atlanta Biologicals, Flowery Branch, GA), 1% penicillin-streptomycin (Life Technologies, Carlsbad, CA), and 2.5 ug/L basic human fibroblast growth factor (bFGF, R&D Systems, Minneapolis, MN). Cells were then counted and plated at a density of roughly 50,000 cells/cm^2^. Cultures were grown for 17 days at 37 °C and 5% CO_2_. Media changes were performed on day 3 and 10 with visual checks daily to assess confluency and potential contamination.

Endothelial (EA.hy296, ATCC, Manassas, VA), hepatocyte (HEPG2, ATCC, Manassas, VA), and fibroblast (NHDF, ATCC, Manassas, VA) cell lines were also tested as control cell types. These three cells lines were individually grown in media composed of: DMEM (Sigma, St. Louis, MO), 1% penicillin-streptomycin (Life Technologies, Carlsbad, CA), and 10% FBS (Atlanta Biologics, Flowery Branch, GA).

Cell dissociation was performed using trypsin at 80% confluence (Sigma, St. Louis, MO). Cells stocks were made at a concentration of 1M cells/mL in freezing media composed of 90% FBS (Atlanta Biological, Flowery Branch, GA) and 10% DMSO (Sigma, St. Louis, MO). Aliquots were frozen in a controlled manner in a −80 °C freezer for 24 hours and subsequently transferred to the vapor phase liquid nitrogen for long term storage.

### Human Peripheral Blood Mononuclear Cells (PBMC) Processing

PBMCs were obtained fresh from healthy donors (Massachusetts General Hospital, Boston, MA). Approval for the consented collection of blood from healthy volunteers and the testing of biospecimens was obtained from the Institutional Review Board of Massachusetts General Hospital (Reg. No. 2011B000346). All methods involving these samples were performed in accordance with institutional and biosafety regulations. Whole blood was diluted 1:1 with warm PBS. 35 mL of diluted blood was layered on top of 15 mL of Ficoll (GE Healthcare, Chicago, IL) and centrifuged at 400 g for 30 minutes at 25 °C. The mononuclear layer was carefully transferred to another tube with complete PBMC media and spun at 300 g for 10 minutes at 20 °C. Complete media used in culture was composed of: RPMI 1640 (Sigma, St. Louis, MO), 10% fetal bovine serum (Atlanta Biologicals, Flowery Branch, GA), and 1% penicillin-streptomycin (Life Technologies, Carlsbad, CA).

Cells stocks were made at a concentration of 10 M cells/mL in freezing media composed of 90% FBS and 10% DMSO (Sigma, St. Louis, MO). PBMCs were frozen in a controlled manner in a −80 °C freezer for 24 hours and subsequently transferred to the vapor phase liquid nitrogen for long term storage.

### T Cell Stimulation Assay and MSC Coculture

MSCs and PBMCs were used from frozen stocks for all experiments. Assays were performed in 24-well plates at the specified volume and cell concentrations per experimental condition. MSCs were seeded in Transwell inserts (GBO, Kremsmunster, Austria) and allowed to adhere overnight. To detect cell proliferation, PBMCs were stained with carboxyflourescein succinimidyl ester (CFSE, Sigma, St. Louis, MO) at 2.5uM. T cell activation was achieved through incubation of PBMCs with 10ug/mL of concanavalin A (ConA, Sigma, St. Louis, MO) and 100 ng/mL of hIL-2 (Sigma, St. Louis, MO) for a period of 4 days. Transwell inserts with seeded MSCs were added to the PBMC wells during the experimental period. Flow cytometry was used to assess CFSE dilution associated with proliferation (LSRII, BD Biosciences, Franklin Lakes, NJ).

### Flow Cytometry

Collected cells were washed in washing buffer (PBS containing 2% (vol/vol) FBS and 2 mM EDTA (Sigma, St. Louis, MO) and stained with: anti-CD3 (BV605, Biolegend, Dedham, MA), anti-CD4 (BUV737, BD, Franklin Lakes, NJ), anti-CD8 (V500, BD, Franklin Lakes, NJ), anti-CD38 (BUV395, BD, Franklin Lakes, NJ), anti-CD25 (PE/Cy7, Biolegend, Dedham, MA) for 20 minutes on ice. Cells were washed twice in washing buffer and cell pellets were then resuspended in 100 uL of fixation buffer (Cytofix, BD, Franklin Lakes, NJ) for 20 minutes on ice. Cells were then washed twice in washing buffer and resuspended in 200 uL of washing buffer. All flow cytometry was performed using an LSRII (BD, Franklin Lakes, NJ). Flow cytometry analysis was performed in FlowJo (Tree Star, Ashland, OR; version 8.7.3). Proliferative cells were gated based on their overall or individual number of cell divisions and raw flow cytometry values (P_raw_) for proliferation were normalized to the positive stimulated control that did not have MSCs (P_+_) (Eq. 1).

1 $$Proliferative\,Fold\,Change=\frac{{P}_{raw}}{{P}_{+}}$$

### ELISA

Cytokine analyses were performed through ELISA based methods. Interferon gamma, interleukin-10, interleukin-1 beta, interleukin-6, tumor necrosis factor alpha, interferon alpha, interleukin-12p40, interleukin-12p70, interleukin-17, interleukin-1RA, interleukin-4, and interferon gamma-induced protein 10, were first assessed by multiplex (Milliplex MAP Human Cytokine/Chemokine Magnetic Bead Panel, EMD Millipore, Burlington, MA). We found that interferon gamma an interleukin-6 fully saturated the multiplex signals and subsequently performed separate individual ELISAs for these two cytokines (R&D Systems, Minneapolis, MN). Prostaglandin E2 was also assessed individually (R&D Systems, Minneapolis, MN).

### Pharmacodynamic Modeling

A standard regression model to fit experimental data was employed to extract parameters related to MSC:PBMC dose responsive effects.

2 $$Proliferation=Min+\frac{Max-Min}{1+{10}^{[(LogIC50-IndependentVariable)\ast HillSlope]}}$$

The variables reflect: max (maximum effect), min (minimum effect), logIC50 (log value of the independent variable at 50% effect), and HillSlope (slope of the exponential portion of the curve).

### Statistical Analyses

All numeric and statistical analyses were performed in Prism (GraphPad, La Jolla, CA). Individual tests are described in figure legends depending on the relevant comparative analysis performed for each study. Pharmacologic analyses implemented a non-linear, 4 parameter regression; correlative interpretations used a linear regression; and two-group significance comparisons were performed with a student’s T-test.

## Electronic supplementary material

Supplementary Information
