# Supplementary material for: Therapeutic Delivery Specifications Identified Through Compartmental Analysis of a Mesenchymal Stromal Cell-Immune Reaction
Source: Sci Rep. 2018 May 1;8:6816. doi: 10.1038/s41598-018-24971-2 (PMC5931547; doi:10.1038/s41598-018-24971-2)
Supplement: Supplementary file 1 — Supplementary Information [file 41598_2018_24971_MOESM1_ESM.pdf]

Therapeutic Delivery Specifications Identified Through Compartmental Analysis of a Mesenchymal Stromal Cell-Immune Reaction

Matthew Li[1], Danika Khong[1], Ling-Yee Chin[1], Amy Singleton[1], Biju Parekkadan[1,2,3*]

[1]Center for Surgery, Innovation, and Bioengineering, Department of Surgery, Massachusetts General Hospital, Harvard Medical School and the Shriners Hospitals for Children, Boston, Massachusetts 02114, USA

[2]Harvard Stem Cell Institute, Cambridge, Massachusetts 02138, USA

[3]Department of Biomedical Engineering, Rutgers University, Piscataway, New Jersey 08854, USA

*Correspondence and requests for materials should be addressed to B.P. (biju_parekkadan@hms.harvard.edu; Shriners Hospitals for Children, 51 Blossom Street, Boston, Massachusetts 02114)

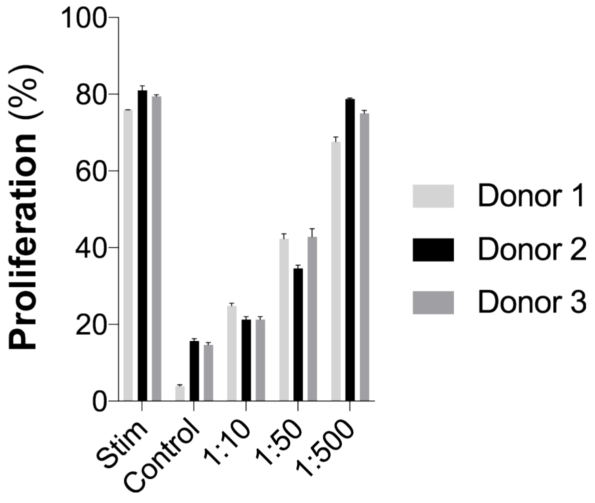

Supplementary Figure 1
Empirical proliferative response of several PBMC donors at several MSC:PBMC ratios. PBMC proliferation was attained through stimulation with ConA and IL2 for a period of 4 days. Proliferation was measured through flow cytometry and CFSE staining; bar graphs represent mean +/- SD of 3 samples. Donor 1 was the donor used for all other experiments in this manuscript. One-way ANOVA performed on this dataset demonstrates non-significant differences between these donors (P=0.40).

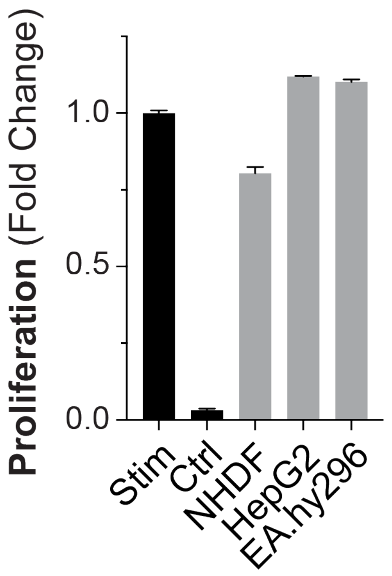

Supplementary Figure 2
Normalized proliferative response of PBMC co-culture with NHDF (dermal fibroblast), HepG2 (liver), and EA.hy296 (endothelial). PBMC proliferation was attained through stimulation with ConA and IL2 for a period of 4 days. Proliferation was measured through flow cytometry and CFSE staining; bar graphs represent mean +/- SD of 3 samples. Non-MSC cells show significantly reduced ability to suppress PBMC proliferation compared to MSCs. HepG2 and EA.hy296 cells interestingly show enhanced proliferation.

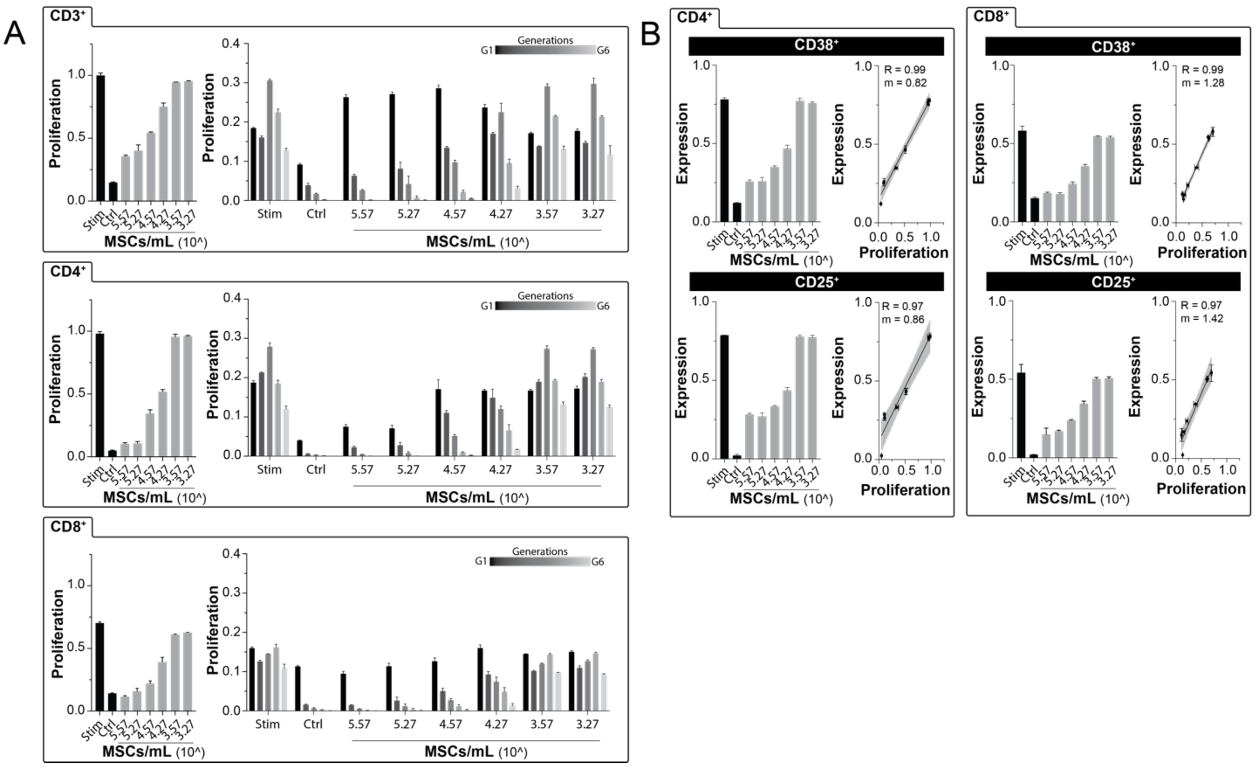

Supplementary Figure 3

The co-culture system can be assessed through generational differences in proliferation, T-Cell specific activation, and cytokine profiling. PBMC proliferation was attained through stimulation with ConA and IL2 for a period of 4 days. Proliferation was measured through flow cytometry and CFSE staining; bar graphs represent mean +/- SD of 3 samples. (*A* $CD3^+$, $CD4^+$, and $CD8^+$ populations exhibit a dose dependent response to MSCs. A maximum of 5 and minimum of 4 generations were detectable in the range of conditions. Values for each generational sample can be found in **Table S3**. (*B*) T-cell phenotype was assessed through two activation markers, CD38 and CD25. Strong correlation and sensitivity between T-cell proliferative levels and marker expression are also apparent. Correlative lines were generated using a linear regression.

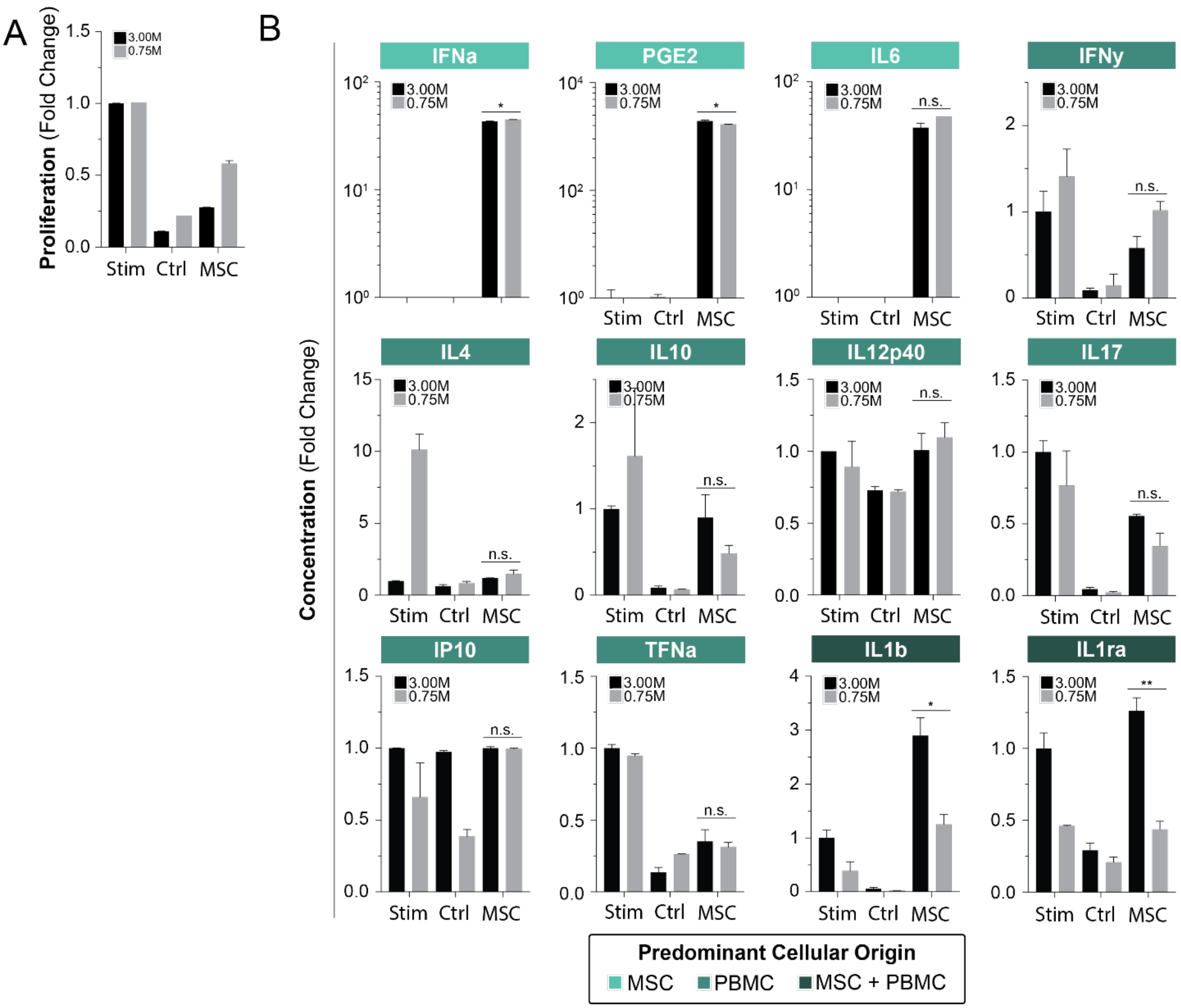

Supplementary Figure 4

PBMC numbers within the microenvironment are important to MSC licensing. PBMC proliferation was attained through stimulation with ConA and IL2 for a period of 4 days. Proliferation was measured through flow cytometry and CFSE staining; bar graphs represent mean +/- SD of 3 samples. (*A*) A 4-fold decrease in PBMC numbers results in a nearly 2-fold decrease in MSC immunosuppressive effectiveness. (*B*) We see a mixed response wherein several pro-inflammatory factors are elevated (such as IFNy) indicating a lack of MSC suppression. We also see suppression in various factors (IL17, TNFa, IL1b) which potentially speaks to sub-optimal levels unable to fully license MSCs resulting from significantly less PBMCs in culture. Two-group significance comparisons were performed with a student's T-test; n.s., no significance; *, $P \leq 0.05$; **, $P \leq 0.01$; *** $P \leq 0.001$.

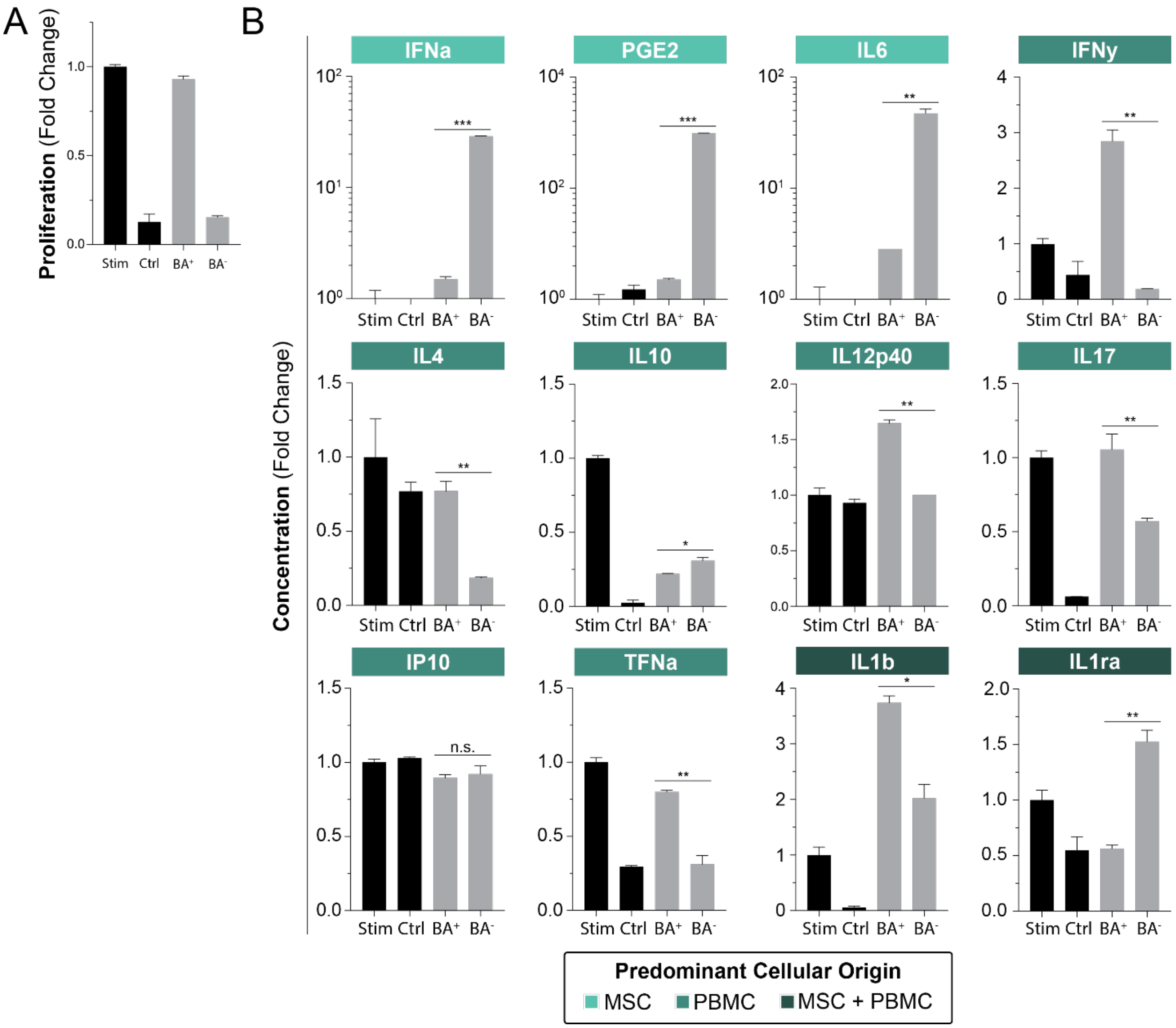

Supplementary Figure 5

Further examination of MSC:PBMC cross communication was explored through the use of Brefeldin A, a protein transport inhibitor. MSCs were treated for 24 hours with Brefeldin A prior to the start of co-culture; +BA indicates with brefeldin A pretreatment, -BA indicates without brefeldin A pretreatment. PBMC proliferation was attained through stimulation with ConA and IL2 for a period of 4 days. Proliferation was measured through flow cytometry and CFSE staining; bar graphs represent mean +/- SD of 3 samples. (*A*) Treatment of MSCs with BA abolished therapeutic function. (*B*) We further find expected results in greatly diminished MSC secreted factors. Significant increases in pro-inflammatory factors are also observed which fall directly in line with MSC immunosuppressive function. Two-group significance comparisons were performed with a student's T-test; n.s., no significance; *, $P \leq 0.05$; **, $P \leq 0.01$; *** $P \leq 0.001$.

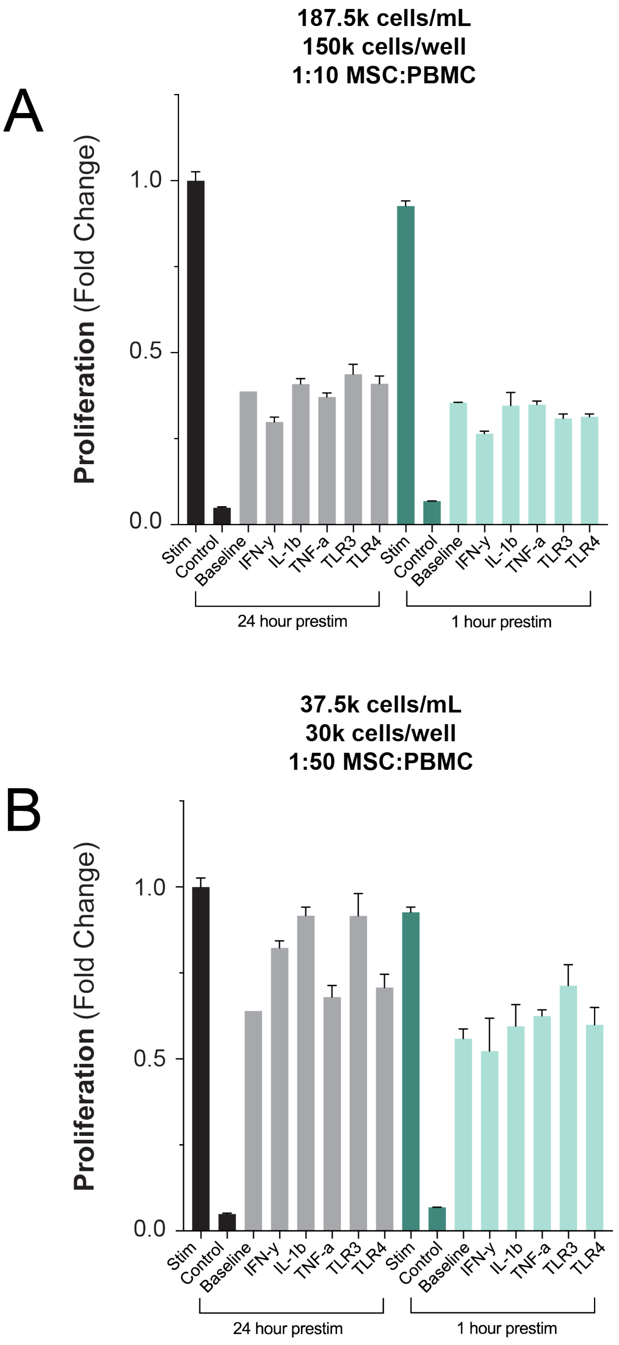

Supplementary Figure 6

Prestimulated MSCs (24 hour and 1 hour) effect on PBMC proliferation. PBMC proliferation was attained through stimulation with ConA and IL2 for a period of 4 days. Proliferation was measured through flow cytometry and CFSE staining; bar graphs represent mean +/- SD of 3 samples. We find no significant enhanced immunosuppressive effects are observed at a known effect and ineffective cell MSC concentration (A & B). In fact, there are clear instances of exacerbated proliferation (IL1b and TLR3 for 24 hour prestim and TLR3 for 1 hour prestim).

SUPPLEMENTARY TABLES

Table S1.
Summary of matched transwell studies for the assessment of ratio, cells/well, and cells/mL as broadly applicable independent variables.

**PBMC** (#)	**MSC Source**	**MSC** (#)	**Volume** (mL)	**Proliferation** (Empirical, %)	**Ref**
300,000	Bone Marrow	100,000<br>30,000<br>10,000	0.6	20<br>67<br>80	[20]
200,000	“ “	20,000	0.2	42	[22]
100,000	“ “	10,000<br>30,000	0.2	50<br>30	[23]
50,000	“ “	15,000	0.2	57	[24]
200,000	“ “	20,000	1.0	55	[25]
100,000	“ “	100,000	0.5	42	[26]
100,000	“ “	20,000<br>20,000	0.5	45<br>65	[27]
100,000	“ “	10,000<br>30,000	0.2	50<br>40	[28]

Table S2.
Regression parameters for ratio, cells/well, and cells/mL used to calculate predicted values from literature.

	**PBMC (#)**	**Max**	**Min**	**HillSlope**	**IC50**	**LogIC50**	**R^2**
**Ratio**	1.5M	90.38	29.87	-3.376	0.01805	-1.744	0.9959
	3.0M	99.79	28.13	-2.109	0.00657	-2.182	0.9935
**MSCs/well**	1.5M	90.38	29.87	-3.376	27075	4.433	0.9959
	3.0M	99.79	28.13	-2.109	19712	4.295	0.9935
**MSCs/mL**	1.5M	90.38	29.87	-3.376	33843	4.529	0.9959
	3.0M	99.79	28.13	-2.109	24641	4.392	0.9935

Table S3.
Proliferative generation values for CD3, CD4, and CD8 populations at various MSC concentrations.

		CD3	CD4	CD8
Generation 1	Stim	0.184	0.187	0.160
	Control	0.091	0.040	0.113
	MSC1	0.263	0.060	0.095
	MSC2	0.271	0.071	0.114
	MSC3	0.286	0.171	0.127
	MSC4	0.237	0.167	0.153
	MSC5	0.171	0.167	0.145
	MSC6	0.177	0.172	0.151
Generation 2	Stim	0.160	0.212	0.126
	Control	0.038	0.006	0.016
	MSC1	0.063	0.023	0.015
	MSC2	0.081	0.036	0.026
	MSC3	0.134	0.110	0.051
	MSC4	0.170	0.149	0.094
	MSC5	0.138	0.190	0.101
	MSC6	0.147	0.202	0.110
Generation 3	Stim	0.305	0.278	0.145
	Control	0.016	0.003	0.006
	MSC1	0.026	0.004	0.005
	MSC2	0.042	0.007	0.011
	MSC3	0.097	0.052	0.027
	MSC4	0.225	0.120	0.064
	MSC5	0.291	0.273	0.121
	MSC6	0.297	0.273	0.127
Generation 4	Stim	0.224	0.184	0.161
	Control	0.002	0.001	0.002
	MSC1	0.002	<0.001	0.001
	MSC2	0.006	<0.001	0.003
	MSC3	0.022	0.066	0.012
	MSC4	0.095	0.066	0.040
	MSC5	0.215	0.193	0.144
	MSC6	0.213	0.189	0.146
Generation 5	Stim	0.127	0.120	0.108
	Control	<0.001	<0.001	<0.001
	MSC1	<0.001	<0.001	<0.001
	MSC2	<0.001	<0.001	<0.001
	MSC3	0.004	0.017	0.003
	MSC4	0.032	0.017	0.008
	MSC5	0.129	0.131	0.098
	MSC6	0.117	0.124	0.093
